# Lower HBV DNA level is associated with more severe liver fibrosis in HBeAg-positive chronic hepatitis B with normal alanine transaminase

**DOI:** 10.1186/s12985-024-02368-0

**Published:** 2024-06-04

**Authors:** Jian Wang, Li Zhu, Zhiyi Zhang, Shaoqiu Zhang, Yifan Pan, Yuanyuan Li, Fei Cao, Chao Jiang, Tao Fan, Ye Xiong, Jiacheng Liu, Yuxin Chen, Shengxia Yin, Xin Tong, Chuanwu Zhu, Xingxiang Liu, Jie Li, Chao Wu, Rui Huang

**Affiliations:** 1 Department of Infectious Diseases, Affiliated Hospital of Medical School, Nanjing Drum Tower Hospital, Nanjing University, No. 321 Zhongshan Road, Nanjing, 210008 Jiangsu China; 2 Institute of Viruses and Infectious Diseases, Nanjing University, Nanjing, Jiangsu China; 3 Department of Infectious Diseases, The Affiliated Infectious Diseases Hospital of Soochow University, Suzhou, Jiangsu China; 4 Department of Infectious Diseases, Nanjing Drum Tower Hospital Clinical College of Nanjing University of Chinese Medicine, Nanjing, Jiangsu China; 5 Department of Infectious Diseases, Nanjing Drum Tower Hospital Clinical College of Nanjing Medical University, Nanjing, Jiangsu China; 6 Department of Infectious Diseases, Nanjing Drum Tower Hospital Clinical College of Jiangsu University, Nanjing, Jiangsu China; 7 Department of Clinical Laboratory, Huai’an No. 4 People’s Hospital, Huai’an, Jiangsu China

**Keywords:** Chronic hepatitis B, Immune-tolerant, HBV DNA, Liver fibrosis, Cirrhosis

## Abstract

**Background:**

The association of hepatitis B virus (HBV) DNA levels and liver fibrosis in chronic hepatitis B (CHB) patients with immune-tolerant phase remains unclear. We explored the association between liver fibrosis and HBV DNA levels in HBeAg-positive CHB patients with normal alanine transaminase (ALT) with relatively high HBV DNA.

**Methods:**

Six hundred and twenty-two HBeAg-positive CHB patients with normal ALT were included. Patients were divided into three categories: low (6 log_10_ IU/mL ≤ HBV DNA < 7 log_10_ IU/mL), moderate (7 log_10_ IU/mL ≤ HBV DNA < 8 log_10_ IU/mL), and high (HBV DNA ≥ 8 log_10_ IU/mL). APRI, FIB-4, transient elastography, or liver biopsy were used to assess liver fibrosis.

**Results:**

The median age of patients was 33.0 years and 57.9% patients were male. 18.8%, 52.1%, and 29.1% of patients had low, moderate, and high HBV DNA levels, respectively. The APRI (0.33 vs. 0.26 vs. 0.26, *P* < 0.001), FIB-4 (1.03 vs. 0.71 vs. 0.68, *P* < 0.001), and LSM values (7.6 kPa vs. 5.6 kPa vs. 5.5 kPa, *P* = 0.086) were higher in low HBV DNA group than other two groups. Low HBV DNA group had higher proportions of significant fibrosis (24.8% vs. 9.9% vs. 3.3%, *P* < 0.001) and cirrhosis (7.7% vs. 2.5% vs. 1.1%, *P* = 0.004) than moderate and high HBV DNA groups. Moderate (OR 3.095, *P* = 0.023) and low (OR 4.968, *P* = 0.003) HBV DNA were independent risk factors of significant fibrosis.

**Conclusion:**

Lower HBV DNA level was associated with more severe liver fibrosis in HBeAg-positive CHB patients with ALT.

**Supplementary Information:**

The online version contains supplementary material available at 10.1186/s12985-024-02368-0.

## Introduction

Chronic hepatitis B virus (HBV) infection is closely related to the development of cirrhosis and hepatocellular carcinoma (HCC) [1]. It is estimated that 296 million individuals are suffering from chronic hepatitis B (CHB) and more than 800,000 people die from this illness each year worldwide [1]. The typical natural history of CHB is a dynamic process and determined by the interplay between host immune response and viral status [2]. The immune-tolerate (IT) or hepatitis B e antigen (HBeAg) positive chronic infection phase usually occurs in the early phase of HBV infection and characterized by hepatitis B e antigen positivity, high serum HBV DNA load, and normal alanine transaminase (ALT) level, suggesting absence of immune-mediated liver damage [2].

Antiviral therapy is generally not recommended for IT subjects according to current guidelines due to the minor liver injury [2, 3]. However, a growing body of evidence suggested that IT phase patients defined by serological parameters had relatively high risk of progressive liver injury, including liver fibrosis, cirrhosis, and HCC [4–6]. Kim et al. reported that untreated IT phase patients had higher risks of severe complications than treated immune active phase patients [4]. A meta-analysis also showed that nearly 20% of IT phase patients had significant liver histologic changes, and antiviral therapy should be initiated immediately for these patients [5]. Therefore, the management strategy for IT patients is in debate.

The associated factors with significant liver fibrosis remains unclear in IT patients. Previous studies reported that age, sex, transaminase, HBV DNA, and HBV variants were related to significant liver fibrosis in IT patients [7–9]. Among these indexes, HBV DNA level has always been a major affecting factor of liver disease progression in patients with CHB [10, 11]. However, few studies evaluated the association between HBV DNA levels and liver fibrosis in CHB patients with IT phase. Thus, the purpose of this study was to explore the association of HBV DNA levels with liver fibrosis in HBeAg-positive CHB patients with normal ALT with relatively high HBV DNA levels by a large multi-center treatment-naïve CHB cohort.

## Methods

### Study population

The multi-center, retrospective study screened 19,911 treatment-naïve patients with CHB between January 2015 and August 2022 at three medical institutions in Jiangsu, China. In the present study, HBeAg positive CHB patients with normal ALT with relatively high HBV DNA levels was included. In detail, patients who met the following inclusion criteria were eligible for this study: (1) positive for hepatitis B surface antigen (HBsAg) over six months; (2) HBeAg positivity; (3) serum HBV DNA levels ≥ 10^6^ IU/mL, (4) normal ALT (< 1 × upper limits of normal [ULN]). The ULNs of ALT were 35 for male and 25 for female, respectively^2^. The excluded criteria were as follows: (1) concurrent with nonalcoholic fatty liver disease, hepatitis C virus infection, hepatitis D virus infection, immune-related liver diseases, hereditary and metabolic liver diseases; (2) HCC or other types of cancer; (3) excessive drinking (> 30 g for men and > 20 g for women per day); (4) insufficiency of clinical data. We did not define patients as IT phase since the phase definition required adequate assessment interval instead of only one timepoint measurement.

The study protocol was approved by the Internal Review Board of Nanjing Drum Tower Hospital (IRB number: 2,008,022) and adhered to the ethical guidelines of the Declaration of Helsinki. A waiver of informed consent was granted by the ethics committees due to a retrospective design. This study was registered under ClinicalTrials.gov (NCT03097952).

### Data collection and definition

Demographic characteristics, medical history, laboratory, and imaging data were retrospectively collected from electronic medical records, including blood routine examination, biochemistry tests, serological markers of HBV, liver histological features, and transient elastography. The HBsAg and HBeAg levels were measured by the ARCHITECT assay (Abbott Gmbh, United States) with the positive threshold values for HBsAg and HBeAg levels of 0.05 IU/ml and 1.0 S/CO, respectively.

Aspartate aminotransferase (AST) to platelet (PLT) ratio index (APRI), fibrosis index based on 4 factors (FIB-4), transient elastography were used to identify significant liver fibrosis and cirrhosis in this study. In addition, some patients underwent liver biopsy and Scheuer scoring system was used to identify liver fibrosis stage (S) [12]. The calculated formulae were as follows: [AST (U/L)/ULN of AST]/PLT (10^9^/L) × 100 for APRI and [age (years) × AST (U/L)]/[PLT (10^9^/L) × (ALT [U/L])^1/2^] for FIB-4 [13, 14]. Individuals who met any of the following criteria were defined as significant liver fibrosis: (1) APRI ≥ 1.5; (2) FIB-4 ≥ 3.25; (3) liver stiffness measurement (LSM) values ≥ 8 kPa [15]; (4) liver histology ≥ S2. Correspondingly, the cirrhosis criteria were as follows: (1) APRI ≥ 2.0; (2) FIB-4 ≥ 6.5; (3) LSM values ≥ 11 kPa [15]; (4) liver histology of fibrosis stage 4.

### Statistical analysis

Continuous and categorical parameters were showed as median (interquartile [IQR]) and number (percentage), respectively. The former were compared by independent-group t-tests (normal distribution) or Mann-Whitney U tests (abnormal distribution), while the latter were compared by chi-squared test. The correlation between HBV DNA levels and liver fibrosis stages was analyzed by Spearman’s method. The risk factors of significant liver fibrosis were identified by logistic regression analysis. A sensitivity analysis was conducted to validate the correlation of HBV DNA levels with liver fibrosis in patients underwent liver biopsy. *P* < 0.05 was considered statistically significant. All analysis was conducted using Statistical Package for the Social Sciences version 23.0 software program (IBM, Armonk, NY, USA) and R software (version 4.2.0; R Foundation, Vienna, Austria; www.R-project.Org).

## Results

### Clinical features of study population

A total of 19,911 treatment-naïve CHB patients were initially screened. According to exclusion criteria, 19,289 patients were excluded and 622 patients were included for the final analysis. Figure S1 shows the flow chart of patient selection.

Of 622 patients, the median age was 33.0 years and male patients accounted for 57.9%. The median levels of ALT, HBsAg, HBeAg, and HBV DNA were 24.0 U/L, 4.6 log_10_ IU/mL, 3.1 log_10_ S/CO, and 7.7 log_10_ IU/mL, respectively (Table 1). The median values of APRI and FIB-4 were 0.27 and 0.72, respectively (Table 2). The data of transient elastography and liver biopsy were available in 152 patients and 66 patients, respectively. The median value of LSM was 5.7 kPa, and the proportions of patients with biopsy-determined significant fibrosis (≥ S2) were 36.4% (Table 2).

**Table 1:** Comparison of clinical features among different HBV DNA subgroups

Variables	All patients (*n* = 622)	6 ≤ HBV DNA < 7 log_10_ IU/mL (*n* = 117)	7 ≤ HBV DNA < 8 log_10_ IU/mL (*n* = 324)	HBV DNA ≥ 8 log_10_ IU/mL (*n* = 181)	*P* value
Age (yr)	33.0 (29.8, 39.0)	37.0 (31.0, 48.0)	33.0 (29.0, 37.8)	32.0 (29.0, 37.0)	< 0.001
Male (%)	360 (57.9)	82 (70.1)	192 (59.3)	86 (47.5)	< 0.001
PLT (×10^9^/L)	203.5 (166.8, 244.3)	183.0 (135.5, 236.0)	200.0 (169.3, 240.8)	219.0 (179.0, 255.5)	< 0.001
Missing, No.	0	0	0	0	
Neutrophils (×10^9^/L)	3.2 (2.4, 4.2)	3.1 (2.2, 4.3)	3.3 (2.5, 4.2)	3.0 (2.4, 4.0)	0.302
Missing, No.	0	0	0	0	
Lymphocytes (×10^9^/L)	1.8 (1.4, 2.2)	1.7 (1.2, 2.1)	1.8 (1.5, 2.2)	1.8 (1.4, 2.1)	0.021
Missing, No.	0	0	0	0	
ALT (U/L)	24.0 (19.6, 29.2)	25.0 (19.9, 31.0)	24.0 (20.0, 29.0)	22.8 (19.3, 28.4)	0.120
Missing, No.	0	0	0	0	
AST (U/L)	22.0 (19.3, 25.6)	24.0 (20.0, 30.2)	21.8 (19.0, 25.4)	21.0 (19.2, 24.2)	< 0.001
Missing, No.	0	0	0	0	
ALP (U/L)	65.0 (54.4, 79.8)	71.7 (57.2, 92.0)	64.0 (53.9, 76.1)	62.0 (53.6, 76.8)	< 0.001
Missing, No.	16	6	5	5	
GGT (U/L)	17.0 (13.0, 23.0)	21.6 (15.0, 34.3)	16.9 (13.1, 21.8)	15.7 (12.0, 20.6)	< 0.001
Missing, No.	13	3	4	6	
Tbil (µmol/L)	12.1 (8.8, 15.8)	13.0 (9.1, 18.7)	12.2 (9.0, 15.8)	11.6 (8.3, 14.3)	0.011
Missing, No.	6	2	3	1	
ALB (g/L)	44.4 (41.8, 46.0)	42.7 (38.3, 45.4)	44.6 (42.2, 46.0)	44.5 (42.6, 46.2)	< 0.001
Missing, No.	9	3	4	2	
GLB (g/L)	28.4 (25.8, 31.3)	27.9 (25.5, 30.9)	28.3 (25.8, 31.3)	29.2 (26.2, 31.6)	0.229
Missing, No.	15	6	5	4	
HBsAg (log_10_ IU/mL)	4.6 (4.3, 4.8)	3.9 (3.3, 4.5)	4.7 (4.4, 4.8)	4.7 (4.6, 4.9)	< 0.001
Missing, No.	110	26	60	24	
HBeAg (log_10_ S/CO)	3.1 (3.1, 3.2)	2.7 (1.8, 3.1)	3.2 (3.1, 3.2)	3.2 (3.1, 3.2)	< 0.001
Missing, No.	73	19	42	12	
HBV DNA (log_10_ IU/mL)	7.7 (7.3, 8.1)	6.5 (6.3, 6.8)	7.7 (7.4, 7.8)	8.2 (8.1, 8.3)	< 0.001
Missing, No.	0	0	0	0	

ALB, albumin; ALP, alkaline phosphatase; ALT, alanine transaminase; AST, aspartate aminotransferase; HBeAg, hepatitis B e antigen; HBsAg, hepatitis B surface antigen; HBV, hepatitis B virus; GGT, gamma-glutamyl transpeptidase; GLB, globulin; PLT, platelet; Tbil, total bilirubin

**Table 2:** Comparison of liver fibrosis degree among different HBV DNA subgroups

Variables	All patients (*n* = 622)	6 ≤ HBV DNA < 7 log_10_ IU/mL (*n* = 117)	7 ≤ HBV DNA < 8 log_10_ IU/mL (*n* = 324)	HBV DNA ≥ 8 log_10_ IU/mL (*n* = 181)	*P* value
APRI	0.27 (0.22, 0.34)	0.33 (0.24, 0.55)	0.26 (0.22, 0.33)	0.26 (0.21, 0.31)	< 0.001
Significant liver fibrosis (≥ 1.5)	9 (1.4)	5 (4.3)	4 (1.2)	0	0.009
Cirrhosis (≥ 2.0)	4 (0.6)	1 (0.9)	3 (0.9)	0	0.436
Missing, No.	0	0	0	0	
FIB-4	0.72 (0.56, 1.03)	1.03 (0.68, 1.99)	0.71 (0.54, 0.98)	0.68 (0.54, 0.87)	< 0.001
Significant liver fibrosis (≥ 3.25)	25 (4.0)	16 (13.7)	9 (2.8)	0	< 0.001
Cirrhosis (≥ 6.5)	7 (1.2)	5 (4.3)	2 (0.6)	0	0.001
Missing, No.	0	0	0	0	
Liver stiffness (kPa)	5.7 (4.6, 6.8)	7.1 (5.1, 10.1)	5.6 (4.8, 6.8)	5.5 (4.4, 6.4)	0.086
Significant liver fibrosis (≥ 8.0)	19 (12.5)	5 (38.5)	10 (14.1)	4 (5.9)	0.004
Cirrhosis (≥ 11.0)	8 (5.3)	2 (15.4)	4 (5.6)	2 (2.9)	0.180
Missing, No.	470	104	253	113	
Liver biopsy					
Significant liver fibrosis (S ≥ 2)	24 (36.4)	8 (72.7)	14 (34.1)	2 (14.3)	0.009
Cirrhosis (S4)	3 (4.5)	2 (18.2)	1 (2.4)	0	0.055
Missing, No.	556	106	283	167	

APRI, AST to PLT ratio index; FIB-4, fibrosis index based on 4 factors; HBV, hepatitis B virus

### Comparison of clinical features and liver fibrosis among different HBV DNA subgroups

Patients were classified into three categories base on the serum HBV DNA levels: low (6 log_10_ IU/mL ≤ HBV DNA < 7 log_10_ IU/mL), moderate (7 log_10_ IU/mL ≤ HBV DNA < 8 log_10_ IU/mL), and high (HBV DNA ≥ 8 log_10_ IU/mL). The proportion of patients with lower, moderate, and high HBV DNA levels were 18.8%, 52.1%, and 29.1%, respectively.

In aspect of clinical features, patients with low HBV DNA were older (37.0 years vs. 33.0 years vs. 32.0 years, *P* < 0.001) and had higher proportion of male gender (70.1% vs. 59.3% vs. 47.5%, *P* < 0.001), levels of AST (24.0 U/L vs. 21.8 U/L vs. 21.0 U/L, *P* < 0.001), alkaline phosphatase (ALP) (71.7 U/L vs. 64.0 U/L vs. 62.0 U/L, *P* < 0.001), gamma-glutamyl transpeptidase (GGT) (21.6 U/L vs. 16.9 U/L vs. 15.7 U/L, *P* < 0.001), and total bilirubin (Tbil) (13.0 µmol/L vs. 12.2 µmol/L vs. 11.6 µmol/L, *P* = 0.011), while had lower levels of PLT (183.0 × 10^9^/L vs. 200.0 × 10^9^/L vs. 219.0 × 10^9^/L, *P* < 0.001), albumin (ALB) (42.7 g/L vs. 44.6 g/L vs. 44.5 g/L, *P* < 0.011), and HBsAg (3.9 log_10_ IU/mL vs. 4.7 log_10_ IU/mL vs. 4.7 log_10_ IU/mL, *P* < 0.011) compared to patients with moderate, and high HBV DNA (Table 1).

**Fig. 1:**
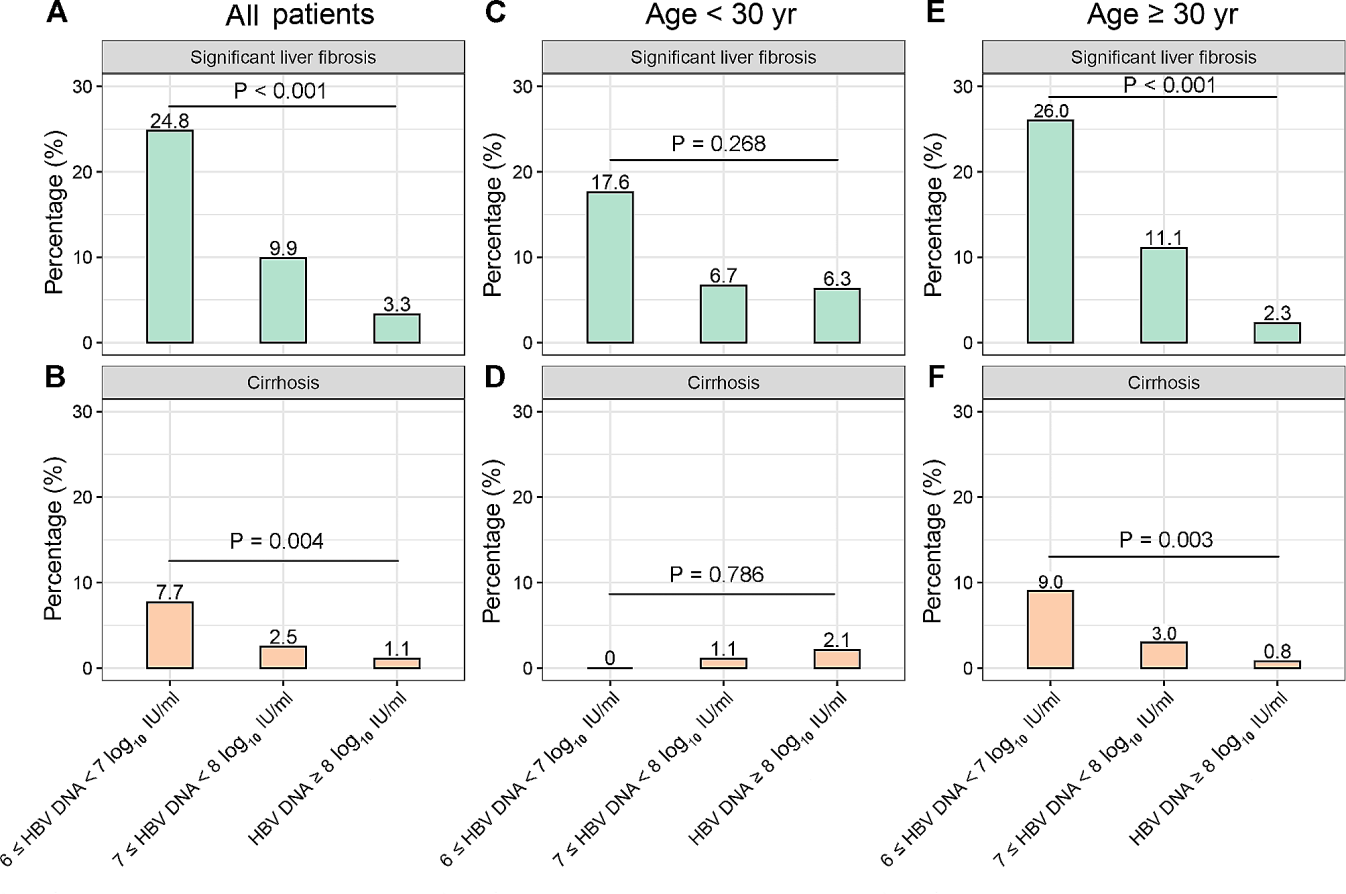
The proportions of significant fibrosis and cirrhosis among different HBV DNA subgroups

With regards to liver fibrosis, the APRI (0.33 vs. 0.26 vs. 0.26, *P* < 0.001), FIB-4 (1.03 vs. 0.71 vs. 0.68, *P* < 0.001), and LSM values (7.1 kPa vs. 5.6 kPa vs. 5.5 kPa, *P* = 0.086) were higher in patients with low HBV DNA than those of patients with moderate, and high HBV DNA. A subgroup analysis was conducted in 66 patients who underwent liver biopsy, which revealed that patients with low HBV DNA had higher proportion of significant fibrosis (72.7% vs. 34.1% vs. 14.3%, *P* = 0.009) and cirrhosis (18.2% vs. 2.4% vs. 0%, *P* = 0.009) than other two groups (Table 2). Overall, patients with low HBV DNA had higher proportion of significant fibrosis (24.8% vs. 9.9% vs. 3.3%, *P* < 0.001) and cirrhosis (7.7% vs. 2.5% vs. 1.1%, *P* = 0.004) compared to patients with moderate and high HBV DNA (Fig. 1A and B). In addition, we also compared the clinical features between patients with and without significant fibrosis, which suggested that patients with significant fibrosis had a lower HBV DNA level (7.1 log_10_ IU/ml vs. 7.8 log_10_ IU/ml, *P* < 0.001) than those without significant fibrosis (Table S1).

### Associated factors of significant liver fibrosis in CHB patients with immune-tolerate phase

Logistic regression analysis was performed to identify associated factors of significant fibrosis in CHB patients with IT phase (Table 3). Of note, several parameters were not included into logistic regression analysis due to multicollinearity, including age, PLT, ALT, and AST. In the univariate analysis, male sex, higher levels of neutrophils, lymphocytes, ALP, GGT, Tbil, low levels of ALB and HBV DNA were associated with significant fibrosis. Low HBV DNA (odds ratio [OR] 4.968, 95% confidence interval [CI] 1.706–14.473, *P* = 0.003) and moderate HBV DNA (OR 3.095, 95% CI 1.165–8.222, *P* = 0.023) remained independent risk factors of significant liver fibrosis in the multivariate analysis.

**Table 3:** Analysis of clinical parameters associated with significant liver fibrosis

Variables	Univariate (OR 95% CI)	*P* value	Multivariate (OR 95% CI)	*P* value
**Sex**				
Female	Reference		Reference	
Male	2.780 (1.528, 5.057)	0.001	1.607 (0.796, 3.243)	0.186
Neutrophils (×10^9^/L)	0.746 (0.609, 0.915)	0.005	0.827 (0.660, 1.037)	0.100
Lymphocytes (×10^9^/L)	0.605 (0.382, 0.957)	0.032	0.950 (0.603, 1.498)	0.826
ALP (U/L)	1.010 (1.003, 1.017)	0.003	1.002 (0.994, 1.011)	0.585
GGT (U/L)	1.032 (1.019, 1.045)	< 0.001	1.014 (1.003, 1.027)	0.017
Tbil (µmol/L)	1.091 (1.054, 1.131)	< 0.001	1.048 (1.009, 1.089)	0.015
ALB (g/L)	0.880 (0.837, 0.926)	< 0.001	0.943 (0.886, 1.004)	0.065
GLB (g/L)	0.990 (0.930, 1.054)	0.762		
**HBV DNA **(log_10_ IU/mL)				
> 8	Reference		Reference	
7–8	3.196 (1.310, 7.798)	0.011	3.095 (1.165, 8.222)	0.023
6–7	9.612 (3.847, 24.013)	< 0.001	4.968 (1.706, 14.473)	0.003

ALB, albumin; ALP, alkaline phosphatase; CI, confidence interval; HBV, hepatitis B virus; GGT, gamma-glutamyl transpeptidase; GLB, globulin; Tbil, total bilirubin; OR, odds ratio

### Association of HBV DNA levels with liver fibrosis in different age subgroups

Further analysis was conducted in patients with age < 30 years and age ≥ 30 years (Table 4). The proportion of patients with low HBV DNA, moderate HBV DNA, and high HBV DNA were 11.0%, 58.1%, and 31.0% in patients age < 30 years, respectively. The values of APRI, FIB-4, LSM, and the proportion of significant liver fibrosis and cirrhosis were comparable among three groups (Fig. 1C and D). Eight patients underwent liver biopsy in age < 30 years group. Although more patients with low HBV DNA (75.0%) had significant liver fibrosis than patients with moderate HBV DNA (25.0%) and high HBV DNA (20.0%), the differences were not statistically significant (*P* = 0.129). However, for patients age ≥ 30 years, those with low HBV DNA had higher values of APRI (0.35 vs. 0.27 vs. 0.25, *P* < 0.001), FIB-4 (1.16 vs. 0.79 vs. 0.71, *P* < 0.001), and LSM (7.4 vs. 5.5 vs. 5.5, *P* = 0.033) compared to those with moderate and high HBV DNA levels. Patients with low HBV DNA also had the highest proportion of significant liver fibrosis (26.0% vs. 11.1% vs. 2.3%, *P* < 0.001) and cirrhosis (9.0% vs. 3.0% vs. 0.8%, *P* = 0.003) than those with moderate HBV DNA and high HBV DNA (Fig. 1E and F). Moreover, we also conduct similar subgroup analysis between patients with age < 35 years and age ≥ 35 years, age < 40 years and age ≥ 40 years. Similar results were observed (Table S2 and Figure S2).

**Table 4:** Comparison of liver fibrosis degree in different age subgroups

Variables	All patients (*n* = 622)	6 ≤ HBV DNA < 7 log_10_ IU/mL (*n* = 117)	7 ≤ HBV DNA < 8 log_10_ IU/mL (*n* = 324)	HBV DNA ≥ 8 log_10_ IU/mL (*n* = 181)	*P* value
**Age < 30 year, No.**	155	17	90	48	
**APRI**	0.26 (0.21, 0.32)	0.24 (0.19, 0.34)	0.26 (0.21, 0.33)	0.26 (0.20, 0.30)	0.661
Significant liver fibrosis (≥ 1.5)	2 (1.3)	0	2 (2.2)	0	0.481
Cirrhosis (≥ 2.0)	1 (0.6)	0	1 (1.1)	0	0.695
Missing, No.	0	0	0	0	
**FIB-4**	0.55 (0.44, 0.70)	0.54 (0.40, 0.81)	0.58 (0.43, 0.72)	0.52 (0.44, 0.69)	0.625
Significant liver fibrosis (≥ 3.25)	1 (0.6)	0	1 (1.1)	0	0.695
Cirrhosis (≥ 6.5)	0	0	0	0	-
Missing, No.	0	0	0	0	
**Liver stiffness (kPa)**	5.7 (4.4, 6.6)	-	5.8 (4.8, 6.5)	5.4 (4.2, 6.8)	0.267
Significant liver fibrosis (≥ 8.0)	2 (4.5)	0	0	2 (10.0)	0.284
Cirrhosis (≥ 11.0)	1 (2.3)	0	0	1 (5.0)	0.541
Missing, No.	111	16	67	28	
**Liver biopsy**					
Significant liver fibrosis (S ≥ 2)	8 (32.0)	3 (75.0)	4 (25.0)	1 (20.0)	0.129
Cirrhosis (S4)	0	0	0	0	-
Missing, No.	130	13	74	43	
**Age ≥ 30 year, No.**	467	100	234	133	
**APRI**	0.27 (0.22, 0.35)	0.35 (0.26, 0.61)	0.27 (0.22, 0.33)	0.25 (0.22, 0.31)	< 0.001
Significant liver fibrosis (≥ 1.5)	7 (1.5)	5 (5.0)	2 (0.9)	0	0.004
Cirrhosis (≥ 2.0)	3 (0.6)	1 (1.0)	2 (0.9)	0	0.542
Missing, No.	0	0	0	0	
**FIB-4**	0.80 (0.62, 1.11)	1.16 (0.73, 2.50)	0.79 (0.61, 1.04)	0.71 (0.61, 0.98)	< 0.001
Significant liver fibrosis (≥ 3.25)	24 (5.1)	16 (16.0)	8 (3.4)	0	< 0.001
Cirrhosis (≥ 6.5)	7 (1.5)	5 (5.0)	2 (0.9)	0	0.004
Missing, No.	0	0	0	0	
**Liver stiffness (kPa)**	5.6 (4.6, 7.0)	7.4 (5.8, 10.4)	5.5 (4.7, 7.2)	5.5 (4.5, 6.2)	0.033
Significant liver fibrosis (≥ 8.0)	17 (15.7)	5 (41.7)	10 (20.8)	2 (4.2)	0.003
Cirrhosis (≥ 11.0)	7 (6.5)	2 (16.7)	4 (8.3)	1 (2.1)	0.145
Missing, No.	359	88	186	85	
**Liver biopsy**					
Significant liver fibrosis (S ≥ 2)	16 (39.0)	5 (71.4)	10 (40.0)	1 (11.1)	0.049
Cirrhosis (S4)	3 (7.3)	2 (28.6)	1 (4.0)	0	0.056
Missing, No.	426	93	209	124	

APRI, AST to PLT ratio index; FIB-4, fibrosis index based on 4 factors; HBV, hepatitis B virus

Logistic regression analysis was performed to identify associated factors of significant fibrosis in patients with age < 30 years and age ≥ 30 years, respectively (Table 5). In age < 30 years group, HBV DNA levels were not associated with significant fibrosis. However, moderate HBV DNA (OR 6.487, 95% CI 1.489–28.255, *P* = 0.013) and low HBV DNA (OR 8.618, 95% CI 1.836–40.458, *P* = 0.006) were independent risk factors of significant fibrosis compared to high HBV DNA in group aged ≥ 30 years.

**Table 5:** Analysis of clinical parameters associated with significant liver fibrosis in different age subgroups

Variables	Univariate (OR 95% CI)	*P* value	Multivariate (OR 95% CI)	*P* value
**Age < 30 yr**				
**Sex**				
Female	Reference			
Male	1.763 (0.508, 6.119)	0.372		
Neutrophils (×10^9^/L)	0.972 (0.618, 1.529)	0.902		
Lymphocytes (×10^9^/L)	1.153 (0.417, 3.189)	0.784		
ALP (U/L)	1.002 (0.990, 1.013)	0.788		
GGT (U/L)	1.032 (0.947, 1.125)	0.470		
Tbil (µmol/L)	1.063 (0.969, 1.167)	0.195		
ALB (g/L)	0.899 (0.760, 1.065)	0.219		
GLB (g/L)	0.967 (0.840, 1.113)	0.640		
**HBV DNA **(log_10_ IU/mL)				
> 8	Reference			
7–8	1.071 (0.256, 4.488)	0.925		
6–7	3.214 (0.582, 17.754)	0.181		
**Age ≥ 30 yr**				
**Sex**				
Female	Reference		Reference	
Male	3.103 (1.559, 6.180)	0.001	1.693 (0.738, 3.883)	0.214
Neutrophil (×10^9^/L)	0.710 (0.561, 0.897)	0.004	0.775 (0.594, 1.011)	0.060
Lymphocyte (×10^9^/L)	0.531 (0.318, 0.886)	0.015	0.839 (0.504, 1.399)	0.502
ALP (U/L)	1.027 (1.015, 1.039)	< 0.001	1.009 (0.994, 1.025)	0.245
GGT (U/L)	1.032 (1.019, 1.045)	< 0.001	1.014 (1.001, 1.026)	0.035
Tbil (µmol/L)	1.096 (1.054, 1.140)	< 0.001	1.044 (1.003, 1.087)	0.034
ALB (g/L)	0.881 (0.835, 0.930)	< 0.001	0.958 (0.894, 1.026)	0.220
GLB (g/L)	0.995 (0.927, 1.067)	0.884		
**HBV DNA **(log_10_ IU/mL)				
> 8	Reference		Reference	
7–8	5.417 (1.607, 18.256)	0.006	6.487 (1.489, 28.255)	0.013
6–7	15.225 (4.456, 52.021)	< 0.001	8.618 (1.836, 40.458)	0.006

ALB, albumin; ALP, alkaline phosphatase; CI, confidence interval; HBV, hepatitis B virus; GGT, gamma-glutamyl transpeptidase; GLB, globulin; Tbil, total bilirubin; OR, odds ratio

Further correlation analysis was conducted between HBV DNA levels and APRI, FIB-4, LSM values, and fibrosis stages (Table S3). Overall, HBV DNA levels were negatively associated with APRI (*r* =-0.226, *P* < 0.001), FIB-4 (*r* =-0.244, *P* < 0.001), LSM (*r* =-0.158, *P* = 0.051), and fibrosis stages (*r* =-0.287, *P* = 0.020). Similar results were observed in patients with age ≥ 35 years and ≥ 40 years.

### Subgroup analysis of clinical features and liver fibrosis in immune-tolerate patients diagnosed by at least two tests

A total of 134 patients met the IT phase criteria with at least two tests taken more than three months apart within a 1-year period, including 15 patients in low HBV DNA group, 73 patients in moderate HBV DNA group, and 46 patients in high HBV DNA group. The age, sex, PLT, ALT, and AST levels were comparable, while HBsAg level (4.4 log_10_ IU/mL vs. 4.7 log_10_ IU/mL vs. 4.8 log_10_ IU/mL, *P* < 0.001) showed an increasing trend in patients with low, moderate, and high HBV DNA levels. With regards to liver fibrosis, patients in low HBV DNA group had higher values of APRI (0.30 vs. 0.25 vs. 0.26, *P* = 0.056), FIB-4 (0.88 vs. 0.67 vs. 0.71, *P* = 0.034), and LSM (6.3 vs. 5.8 vs. 5.5, *P* = 0.814) than patients in moderate and high HBV DNA group (Table S4). In addition, we also compared the clinical features between HBeAg-positive CHB patients with normal ALT with relatively high HBV DNA levels with at least two measurements and those with only one-time measurement (Table S5). The results suggested that patients with at least two measurements had higher HBsAg (4.7 log_10_ IU/ml vs. 4.6 log_10_ IU/ml, *P* < 0.001) and HBV DNA (7.8 log_10_ IU/ml vs. 7.7 log_10_ IU/ml, *P* = 0.002) levels than those only one-time measurement, while age and liver fibrosis degree were comparable between two groups.

## Discussion

In this multi-center study, we analyzed the association of HBV DNA levels with liver fibrosis in HBeAg-positive CHB patients with normal ALT with relatively high HBV DNA levels. The results indicated that patients with low HBV DNA level had more severe liver fibrosis compared to those with higher HBV DNA. A lower HBV DNA level was identified as a risk factor for significant liver fibrosis in HBeAg-positive CHB patients with normal ALT with relatively high HBV DNA levels.

The definition of IT phase is commonly based on serological markers including HBeAg positivity, normal ALT and high HBV DNA levels, which had minimal or no immune-mediated liver injury. Hui et al. analyzed 57 IT phase patients and 66.7% of patients had mild liver fibrosis, while none of patients had significant liver fibrosis. However, a growing body of evidence showed that although HBV-mediated immune response in IT phase is mild, substantial proportion of patients may have significant liver fibrosis. A meta-analysis, which included 9,377 CHB patients with IT phase underwent liver biopsy, revealed that nearly one third of patients had significant fibrosis or more severe fibrosis [16]. Yoo et al. reported that nearly 70% of patients who belonged to serological IT phase were not in true histologic IT phase [6]. Our previous study also demonstrated that over 30% of CHB patients with serological IT phase had significant liver fibrosis [17]. In addition, the long-term prognosis of CHB patients in the IT phase is generally favorable, with a low risk of disease progression [2, 3]. A meta-analysis revealed that HBeAg-positive CHB patients in the untreated IT phase and those in the antiviral-treated immune active phase had comparable clinical outcomes, including the development of HCC and death [18]. On the contrary, Kim et al. found that untreated CHB patients with IT phase had higher risk of HCC and death/transplantation than treated immune-active phase patients [4]. However, the mean age of IT phase patients was 38 years in this study and many patients need antiviral treatment according to current guidelines [4].

The inconsistent results of histological change and prognosis in IT phase patients may be due to the discrepancies of clinical features in previous studies, especially different HBV DNA levels. Xie et al. reported that 6.7 log_10_ IU/mL was the optimal threshold value of HBV DNA to identify significant fibrosis, and no patient had significant fibrosis in HBeAg positive CHB patients with HBV DNA > 8.5 log_10_ IU/mL [19]. Our previous study also found that patients with low HBV DNA level (5–7 log_10_ IU/mL) had higher proportion of significant inflammation in CHB patients with normal ALT [20]. Another study reported that HBV DNA levels of 6–7 log_10_ IU/mL in HBeAg positive CHB patients with normal ALT had a higher risk of HCC than patients with higher HBV DNA levels [21]. In the present study, we also demonstrated that HBV DNA levels were negatively associated with liver fibrosis, suggesting that lower HBV DNA levels may be associated with high risk of significant liver fibrosis in HBeAg-positive CHB patients with normal ALT with relatively high HBV DNA levels. However, high level of HBV DNA has always been regarded as a stimulative factor of adverse outcomes in patients with CHB [10, 11]. The potential mechanism behind the contradictory results of HBV DNA impacts on disease progression in CHB patients remains unclear. HBV variants may be one of the main causes of adverse outcomes in CHB patients with IT phase, including liver fibrosis, cirrhosis, and HCC [22, 23]. Yuen et al. found that basal core promoter and hepatitis B X gene variants were independently associated with lower levels of HBV DNA in IT phase patients, and increased HBV diversity was related to older age and lower HBV DNA [9]. In addition, patients with low HBV DNA levels may exist activated anti-HBV immune response, as reflected by higher ALT, AST, ALP, GGT, and Tbil levels in these patients in the present study. Therefore, patients with low HBV DNA might not be actually IT phase, who were more likely immune active phase but with normal ALT, or in the phase changing from IT to immune active phase. Liver biopsy is required to identify patients with significant histological change and initiate antiviral therapy to prevent adverse outcomes in IT phase patients with low HBV DNA levels.

Given the significant association between HBV DNA level and liver fibrosis in early phase of HBV infection, reconsideration of threshold value of HBV DNA is necessary to the definition of serological IT phase. The definition of IT phase as high HBV DNA of more than 10^6^ IU/ml in AASLD guideline is challenged according our results and previous studies [2]. Although higher HBV DNA of 10^7^ IU/ml for IT phase is defined in European Association for the Study of the Liver guideline, patients with HBV DNA of 10^7^-10^8^ IU/ml remain had more severe liver fibrosis than patients with HBV DNA ≥ 10^8^ IU/ml in this study [3]. Patients with lower HBV DNA levels may be not in the “true” IT phase of CHB. Thus, the optimal threshold value of HBV DNA for the definition of IT phase needs to be confirmed in future study.

Several limitations need to be considered for this study. First, the majority of patients before study entry had a single-time point measurement in this study. Patients with low HBV DNA may be progressive toward the immune clearance phase. However, as a sensitive analysis, patients with at least two tests taken more than three months apart within a 1-year period were included and similar results were found. Second, we did not compare the longitudinal progression of liver fibrosis among patients with different HBV DNA levels due to the retrospective design. In addition, the difference of disease progression in patients HBeAg-positive CHB patients with normal ALT with relatively high HBV DNA levels by a single-time test and at least two tests were unclear. Thus, the results need validation by further prospective and longitudinal studies. Third, the degree of liver fibrosis was largely based on noninvasive measurements and most patients were lack of liver biopsy data. However, a sensitive analysis showed similar results in patients underwent liver biopsy. Fourth, we did not analyze the association between HBsAg and liver fibrosis because quantitative data of HBsAg levels were not available in a substantial proportion (17.7%) of patients. Last, HBV genotype data were not available and the HBV mutations were not detected in our study. Thus, the association of HBV genotypes and viral diversity with HBV DNA levels and liver fibrosis progression requires further investigation.

In conclusion, lower HBV DNA level was associated with more severe liver fibrosis in HBeAg-positive CHB patients with normal ALT with relatively high HBV DNA levels, especially for patients older than 30 years. Since a greater proportion of significant fibrosis was observed in HBeAg-positive CHB patients with normal ALT and lower HBV DNA levels, especially in older age groups, these subjects should adhere to the guidelines’ suggestion to receive fibrosis assessment to identify who is eligible for antiviral therapy.

### Electronic supplementary material

Below is the link to the electronic supplementary material.

Supplementary Material 1

## Data Availability

The data that support the study findings are available upon reasonable request from the corresponding authors (RH and CW).

## Abbreviations

ALP: alkaline phosphatase
ALT: alanine transaminase
APRI: AST to PLT ratio index
AST: aspartate aminotransferase
CHB: chronic hepatitis B
CI: confidence interval
FIB-4: fibrosis index based on 4 factors
HBV: hepatitis B virus
HBeAg: hepatitis B e antigen
HBsAg: hepatitis B surface antigen
HCC: hepatocellular carcinoma
IQR: interquartile
IT: immune-tolerate
GGT: gamma-glutamyl transpeptidase
OR: odds ratio
PLT: platelet
Tbil: total bilirubin
ULN: upper limits of normal
