# Supplementary material for: Lower HBV DNA level is associated with more severe liver fibrosis in HBeAg-positive chronic hepatitis B with normal alanine transaminase
Source: Virol J. 2024 Jun 4;21:127. doi: 10.1186/s12985-024-02368-0 (PMC11151594; doi:10.1186/s12985-024-02368-0)

**Supplementary Material**

**Lower HBV DNA level is associated more severe liver fibrosis in HBeAg-positive chronic hepatitis B with normal alanine transaminase**

**Table S1.** Comparison of clinical features between patients with and without significant fibrosis.

**Table S2.** Comparison of liver fibrosis degree in different age subgroups.

**Table S3**. Spearman correlation analysis of HBV DNA levels with liver fibrosis.

**Table S4.** Comparison of liver fibrosis degrees among different HBV DNA subgroups for HBeAg-positive CHB patients with normal ALT with one measurement and at least two measurements.
**Table S5.** Comparison of clinical features of HBeAg-positive CHB patients with normal ALT with one measurement and at least two measurements.

**Figure S1.** Flow chart of patient selection.

**Figure S2.** Comparison of significant fibrosis and cirrhosis proportions among different HBV DNA subgroups in patients with different age.

**Table S1.** Comparison of clinical features between patients with and without significant fibrosis.

| **Variables** | **Without significant fibrosis** (n=555) | **With significant fibrosis (n=67)** | **P value** |
| --- | --- | --- | --- |
| Age (yr) | 33.0 (29.0, 38.0) | 41.0 (31.0, 54.0) | <0.001 |
| Male (%) | 308 (55.5) | 52 (77.6) | 0.001 |
| PLT (×10^9^/L) | 208.0 (173.0, 249.0) | 150.0 (88.0, 199.0) | <0.001 |
| Neutrophils (×10^9^/L) | 3.2 (2.4, 4.2) | 3.0 (2.0, 3.7) | 0.009 |
| Lymphocytes (×10^9^/L) | 1.8 (1.5, 2.2) | 1.6 (1.2, 2.2) | 0.025 |
| ALT (U/L) | 23.8 (19.4, 29.0) | 25.6 (21.0, 31.0) | 0.075 |
| AST (U/L) | 21.7 (19.0, 25.0) | 26.0 (20.9, 43.0) | <0.001 |
| ALP (U/L) | 63.6 (54.0, 76.8) | 82.0 (62.6, 96.3) | <0.001 |
| GGT (U/L) | 16.5 (12.7, 22.0) | 20.5 (15.7, 51.8) | <0.001 |
| Tbil (μmol/L) | 11.8 (8.5, 15.3) | 15.8 (11.9, 22.0) | <0.001 |
| ALB (g/L) | 44.4 (42.1, 46.0) | 42.9 (38.1, 45.8) | 0.004 |
| GLB (g/L) | 28.4 (25.9, 31.3) | 28.0 (25.6, 31.9) | 0.610 |
| HBsAg (log_10_ IU/mL) | 4.7 (4.4, 4.8) | 4.3 (3.2, 4.6) | <0.001 |
| HBeAg (log_10_ S/CO) | 3.2 (3.1, 3.2) | 2.7 (1.9, 3.2) | <0.001 |
| HBV DNA (log_10_ IU/mL) | 7.8 (7.3, 8.1) | 7.1 (6.5, 7.7) | <0.001 |
| HBV DNA range |  |  | <0.001 |
| 6-7 log_10_ IU/mL | 88 (15.9) | 29 (43.3) |  |
| 7-8 log_10_ IU/mL | 292 (52.6) | 32 (47.8) |  |
| ≥8 log_10_ IU/mL | 175 (31.5) | 6 (9.0) |  |

ALB, albumin; ALP, alkaline phosphatase; ALT, alanine transaminase; AST, aspartate aminotransferase; HBeAg, hepatitis B e antigen; HBsAg, hepatitis B surface antigen; HBV, hepatitis B virus; GGT, gamma-glutamyl transpeptidase; GLB, globulin; PLT, platelet; Tbil, total bilirubin.

**Table S2.** Comparison of liver fibrosis degree in different age subgroups.

| **Variables** | **All patients (n=622)** | **6** ≤**HBV DNA** <7 log_10_ IU/mL (n=117) | 7 ≤HBV DNA <8 log_10_ IU/mL (n=324) | **HBV DNA ≥8 log_10_ IU/mL (n=181)** | **P value** |
| --- | --- | --- | --- | --- | --- |
| **Age<35 yr and ≥35 yr** |  |  |  |  |  |
| **Age <35 yr, No.** | **376** | **48** | **206** | **122** |  |
| APRI | 0.25 (0.21, 0.31) | 0.24 (0.19, 0.33) | 0.25 (0.21, 0.32) | 0.26 (0.20, 0.30) | 0.816 |
| Significant liver fibrosis (≥1.5) | 2 (0.5) | 0 | 2 (1.0) | 0 | 0.436 |
| Cirrhosis (≥2.0) | 1 (0.3) | 0 | 1 (0.5) | 0 | 0.661 |
| Missing, No. | 0 | 0 | 0 | 0 | - |
| FIB-4 | 0.62 (0.50, 0.78) | 0.69 (0.51, 0.84) | 0.62 (0.49, 0.78) | 0.62 (0.51, 0.75) | 0.694 |
| Significant liver fibrosis (≥3.25) | 1 (0.3) | 0 | 1 (0.5) | 0 | 0.661 |
| Cirrhosis (≥6.5) | 0 | 0 | 0 | 0 | - |
| Missing, No. | 0 | 0 | 0 | 0 | - |
| Liver stiffness (kPa) | 5.5 (4.4, 6.6) | 6.2 (3.3, -z) | 5.6 (4.4, 6.6) | 5.5 (4.4, 6.5) | 0.965 |
| Significant liver fibrosis (≥8.0) | 9 (8.9) | 0 | 5 (10.6) | 4 (7.8) | 0.764 |
| Cirrhosis (≥11.0) | 3 (3.0) | 0 | 1 (2.1) | 2 (3.9) | 0.832 |
| Missing, No. | 275 | 45 | 159 | 71 | - |
| Liver biopsy |  |  |  |  |  |
| Significant liver fibrosis (S ≥2) | 15 (37.5) | 5 (83.3) | 8 (33.3) | 2 (20.0) | 0.032 |
| Cirrhosis (S4) | 1 (2.5) | 1 (16.7) | 0 | 0 | 0.055 |
| Missing, No. | 336 | 42 | 182 | 112 | - |
| **Age ≥35 yr, No.** | 246 | 69 | 118 | 59 |  |
| APRI | 0.30 (0.23, 0.42) | 0.42 (0.31, 0.89) | 0.29 (0.23, 0.39) | 0.25 (0.21, 0.32) | <0.001 |
| Significant liver fibrosis (≥1.5) | 7 (2.8) | 5 (7.2) | 2 (1.7) | 0 | 0.028 |
| Cirrhosis (≥2.0) | 3 (1.2) | 1 (1.4) | 2 (1.7) | 0 | 0.613 |
| Missing, No. | 0 | 0 | 0 | 0 | - |
| FIB-4 | 1.03 (0.74, 1.54) | 1.76 (1.04, 3.06) | 0.99 (0.77, 1.35) | 0.80 (0.68, 1.10) | <0.001 |
| Significant liver fibrosis (≥3.25) | 24 (9.8) | 16 (23.2) | 8 (6.8) | 0 | <0.001 |
| Cirrhosis (≥6.5) | 7 (2.8) | 5 (7.2) | 2 (1.7) | 0 | 0.028 |
| Missing, No. | 0 | 0 | 0 | 0 | - |
| Liver stiffness (kPa) | 6.0 (4.8, 7.4) | 7.7 (5.4, 11.7) | 6.1 (4.9, 7.7) | 5.5 (4.6, 6.2) | 0.076 |
| Significant liver fibrosis (≥8.0) | 10 (19.6) | 5 (50.0) | 5 (20.8) | 0 | 0.007 |
| Cirrhosis (≥11.0) | 5 (9.8) | 2 (20.0) | 3 (12.5) | 0 | 0.200 |
| Missing, No. | 195 | 59 | 94 | 42 | - |
| Liver biopsy |  |  |  |  |  |
| Significant liver fibrosis (S ≥2) | 9 (34.6) | 3 (60.0) | 6 (35.3) | 0 | 0.170 |
| Cirrhosis (S4) | 2 (7.7) | 1 (20.0) | 1 (5.9) | 0 | 0.477 |
| Missing, No. | 220 | 64 | 101 | 55 | - |
| **Age<40 yr and ≥40 yr** |  |  |  |  |  |
| **Age <40 yr, No.** | 475 | 65 | 260 | 150 |  |
| APRI | 0.26 (0.21, 0.32) | 0.27 (0.21, 0.34) | 0.25 (0.21, 0.32) | 0.26 (0.21, 0.30) | 0.297 |
| Significant liver fibrosis (≥1.5) | 3 (0.6) | 0 | 3 (1.2) | 0 | 0.287 |
| Cirrhosis (≥2.0) | 2 (0.4) | 0 | 2 (0.8) | 0 | 0.436 |
| Missing, No. | 0 | 0 | 0 | 0 | - |
| FIB-4 | 0.66 (0.52, 0.84) | 0.70 (0.54, 0.94) | 0.65 (0.52, 0.83) | 0.65 (0.52, 0.80) | 0.249 |
| Significant liver fibrosis (≥3.25) | 2 (0.4) | 0 | 2 (0.8) | 0 | 0.436 |
| Cirrhosis (≥6.5) | 1 (0.2) | 0 | 1 (0.4) | 0 | 0.661 |
| Missing, No. | 0 | 0 | 0 | 0 | - |
| Liver stiffness (kPa) | 5.6 (4.4, 6.7) | 6.7 (4.0, 7.6) | 5.6 (4.5, 6.8) | 5.5 (4.4, 6.5) | 0.643 |
| Significant liver fibrosis (≥8.0) | 11 (9.0) | 0 | 7 (11.5) | 4 (7.0) | 0.570 |
| Cirrhosis (≥11.0) | 4 (3.3) | 0 | 2 (0.8) | 2 (3.5) | 0.930 |
| Missing, No. | 353 | 61 | 199 | 93 | - |
| Liver biopsy |  |  |  |  |  |
| Significant liver fibrosis (S ≥2) | 17 (35.4) | 6 (85.7) | 9 (31.0) | 2 (16.7) | 0.007 |
| Cirrhosis (S4) | 2 (4.2) | 2 (28.6) | 0 | 0 | 0.002 |
| Missing, No. | 427 | 58 | 231 | 138 | - |
| **Age ≥40 yr, No.** | 147 | 52 | 64 | 31 |  |
| APRI | 0.33 (0.24, 0.50) | 0.50 (0.33, 1.13) | 0.33 (0.24, 0.43) | 0.24 (0.21, 0.32) | <0.001 |
| Significant liver fibrosis (≥1.5) | 6 | 5 (9.6) | 1 (1.6) | 0 | 0.040 |
| Cirrhosis (≥2.0) | 2 | 1 (1.9) | 1 (1.6) | 0 | 0.752 |
| Missing, No. | 0 | 0 | 0 | 0 | - |
| FIB-4 | 1.30 (0.94, 2.14) | 2.27 (1.27, 4.59) | 1.16 (0.95, 1.72) | 0.94 (0.68, 1.14) | <0.001 |
| Significant liver fibrosis (≥3.25) | 23 | 16 (30.8) | 7 (10.9) | 0 | <0.001 |
| Cirrhosis (≥6.5) | 6 | 5 (9.6) | 1 (1.6) | 0 | 0.040 |
| Missing, No. | 0 | 0 | 0 | 0 | - |
| Liver stiffness (kPa) | 6.0 (4.9, 8.5) | 8.3 (5.1, 12.6) | 5.8 (5.0, 10.0) | 5.5 (4.6, 6.3) | 0.188 |
| Significant liver fibrosis (≥8.0) | 8 | 5 (55.6) | 3 (30.0) | 0 | 0.019 |
| Cirrhosis (≥11.0) | 4 | 2 (22.2) | 2 (20.0) | 0 | 0.260 |
| Missing, No. | 117 | 43 | 54 | 20 | - |
| Liver biopsy |  |  |  |  |  |
| Significant liver fibrosis (S ≥2) | 7 | 2 (50.0) | 5 (41.7) | 0 | 0.468 |
| Cirrhosis (S4) | 1 | 0 | 1 (8.3) | 0 | 0.767 |
| Missing, No. | 129 | 48 | 52 | 29 | - |

APRI, AST to PLT ratio index; FIB-4, fibrosis index based on 4 factors; HBV, hepatitis B virus.

**Table S3**. Spearman correlation analysis of HBV DNA levels with liver fibrosis.

|  | **All patients** | | **Age <30 yr** | | **Age ≥30 yr** | | **Age <35 yr** | | **Age ≥35 yr** | | **Age <40 yr** | | **Age ≥40 yr** | |
| --- | --- | --- | --- | --- | --- | --- | --- | --- | --- | --- | --- | --- | --- | --- |
|  | **Rho** | **P value** | **Rho** | **P value** | **Rho** | **P value** | **Rho** | **P value** | **Rho** | **P value** | **Rho** | **P value** | **Rho** | **P value** |
| APRI | -0.226 | <0.001 | -0.062 | 0.441 | -0.272 | <0.001 | -0.044 | 0.392 | -0.402 | <0.001 | -0.076 | 0.097 | -0.476 | <0.001 |
| FIB-4 | -0.244 | <0.001 | -0.105 | 0.195 | -0.287 | <0.001 | -0.056 | 0.281 | -0.412 | <0.001 | -0.086 | 0.061 | -0.501 | <0.001 |
| Liver stiffness (kPa) | -0.158 | 0.051 | 0.137 | 0.377 | -0.248 | 0.010 | -0.001 | 0.989 | -0.358 | 0.010 | -0.056 | 0.524 | -0.352 | 0.056 |
| Fibrosis stage | -0.287 | 0.020 | - | - | - | - | - | - | - | - | - | - | - | - |

APRI, AST to PLT ratio index; FIB-4, fibrosis index based on 4 factors.

**Table S4.** Comparison of liver fibrosis degrees among different HBV DNA subgroups for HBeAg-positive CHB patients with normal ALT with at least two measurements.

| **Variables** | **All patients (n=134)** | 6 **≤**HBV DNA <7 log_10_ IU/mL  (n=15) | 7 **≤**HBV DNA <8 log _10_ IU/mL (n=73) | **HBV DNA ≥8 log _10_ IU/mL (n=46)** | **P value** |
| --- | --- | --- | --- | --- | --- |
| Age (yr) | 32.0 (29.0, 37.0) | 35.0 (31.0, 43.0) | 33.0 (29.0, 37.0) | 32.0 (28.0, 36.0) | 0.178 |
| Male (%) | 63 (47.0) | 9 (60.0) | 35 (47.9) | 19 (41.3) | 0.440 |
| PLT (×10^9^/L) | 212.5 (179.8, 244.3) | 189.0 (160.0, 230.0) | 209.0 (181.5, 243.5) | 225.5 (182.0, 256.8) | 0.194 |
| ALT (U/L) | 23.1 (19.1, 27.1) | 25.0 (16.5, 30.0) | 23.9 (19.4, 27.8) | 21.5 (19.1, 25.9) | 0.348 |
| AST (U/L) | 22.1 (19.0, 25.0) | 24.0 (20.1, 27.0) | 21.1 (18.6, 24.2) | 22.9 (19.6, 25.3) | 0.099 |
| HBsAg (log_10_ IU/mL) | 4.7 (4.5, 4.9) | 4.4 (4.1, 4.5) | 4.7 (4.5, 4.9) | 4.8 (4.6, 4.9) | <0.001 |
| HBeAg (log_10_ S/CO) | 3.2 (3.1, 3.2) | 3.1 (2.8, 3.2) | 3.1 (3.1, 3.2) | 3.2 (3.1, 3.2) | 0.254 |
| HBV DNA (log_10_ IU/mL) | 7.8 (7.6, 8.1) | 6.6 (6.3, 6.8) | 7.7 (7.6, 7.9) | 8.2 (8.1, 8.2) | <0.001 |
| APRI | 0.26 (0.22, 0.33) | 0.30 (0.26, 0.44) | 0.25 (0.21, 0.31) | 0.26 (0.22, 0.33) | 0.056 |
| FIB-4 | 0.69 (0.57, 0.97) | 0.88 (0.72, 1.15) | 0.67 (0.55, 0.90) | 0.71 (0.57, 1.02) | 0.034 |
| Liver stiffness (kPa) | 5.6 (4.6, 6.6) | 6.3 (4.9, 7.0) | 5.8 (4.6, 6.8) | 5.5 (4.7, 6.4) | 0.814 |

ALT, alanine transaminase; APRI, AST to PLT ratio index; AST, aspartate aminotransferase; FIB-4, fibrosis index based on 4 factors; HBeAg, hepatitis B e antigen; HBsAg, hepatitis B surface antigen; HBV, hepatitis B virus; PLT, platelet.

**Table S5.** Comparison of clinical features of HBeAg-positive CHB patients with normal ALT with one measurement and at least two measurements.

| **Variables** | **One measurement** (n=488) | **At least two measurements (n=134)** | **P value** |
| --- | --- | --- | --- |
| Age (yr) | 33.0 (30.0, 40.0) | 32.0 (29.0, 37.0) | 0.115 |
| Male (%) | 297 (60.9) | 63 (47.0) | 0.004 |
| PLT (×10^9^/L) | 201.0 (165.0, 244.5) | 212.5 (179.8, 244.3) | 0.066 |
| Neutrophils (×10^9^/L) | 3.2 (2.4, 4.2) | 3.1 (2.4, 4.0) | 0.844 |
| Lymphocytes (×10^9^/L) | 1.8 (1.4, 2.1) | 1.9 (1.5, 2.2) | 0.087 |
| ALT (U/L) | 24.0 (20.0, 29.9) | 23.1 (19.1, 27.1) | 0.033 |
| AST (U/L) | 22.0 (19.4, 25.9) | 22.1 (19.0, 25.0) | 0.777 |
| ALP (U/L) | 66.0 (54.7, 81.0) | 61.4 (53.5, 71.9) | 0.018 |
| GGT (U/L) | 17.4 (13.1, 24.0) | 15.1 (12.5, 20.5) | <0.001 |
| Tbil (μmol/L) | 12.3 (8.6, 16.1) | 11.8 (9.0, 15.2) | 0.466 |
| ALB (g/L) | 44.2 (41.6, 46.0) | 44.6 (42.8, 46.2) | 0.035 |
| GLB (g/L) | 28.2 (25.7, 31.3) | 29.3 (26.2, 31.6) | 0.140 |
| HBsAg (log_10_ IU/mL) | 4.6 (4.3, 4.8) | 4.7 (4.5, 4.9) | <0.001 |
| HBeAg (log_10_ S/CO) | 3.1 (3.0, 3.2) | 3.1 (3.1, 3.2) | 0.407 |
| HBV DNA (log_10_ IU/mL) | 7.7 (7.2, 8.0) | 7.8 (7.6, 8.1) | 0.002 |
| HBV DNA range |  |  | 0.029 |
| 6-7 log_10_ IU/mL | 102 (20.9) | 15 (11.2) |  |
| 7-8 log_10_ IU/mL | 251 (51.4) | 73 (54.5) |  |
| ≥8 log_10_ IU/mL | 135 (27.7) | 46 (34.3) |  |
| APRI | 0.27 (0.22, 0.35) | 0.26 (0.22, 0.33) | 0.212 |
| FIB-4 | 0.73 (0.55, 1.05) | 0.69 (0.57, 0.97) | 0.322 |
| Liver stiffness (kPa) | 5.7 (4.6, 6.9) | 5.6 (4.6, 6.6) | 0.780 |

ALB, albumin; ALP, alkaline phosphatase; ALT, alanine transaminase; AST, aspartate aminotransferase; HBeAg, hepatitis B e antigen; HBsAg, hepatitis B surface antigen; HBV, hepatitis B virus; GGT, gamma-glutamyl transpeptidase; GLB, globulin; PLT, platelet; Tbil, total bilirubin.

**Figure S1.** Flow chart of patient selection.


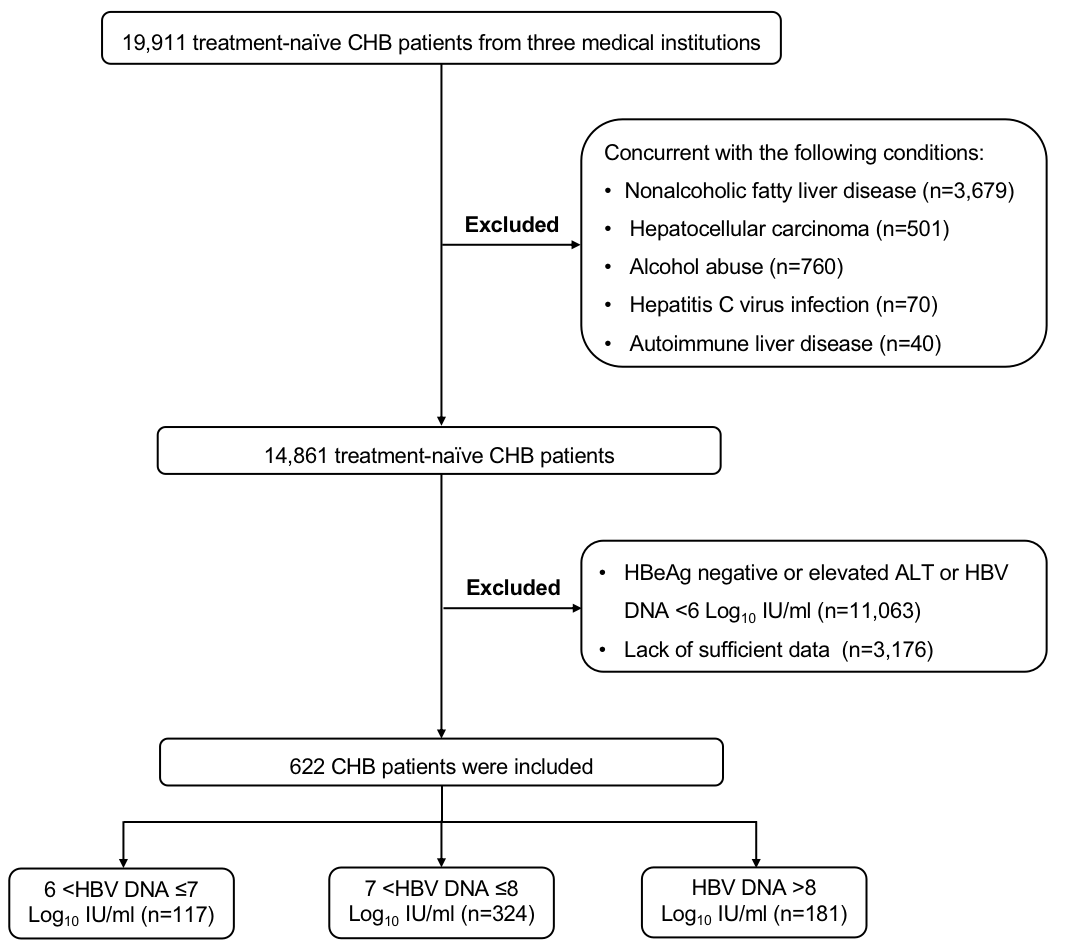


**Figure S2.** Comparison of significant fibrosis and cirrhosis proportions among different HBV DNA subgroups in patients with different age.


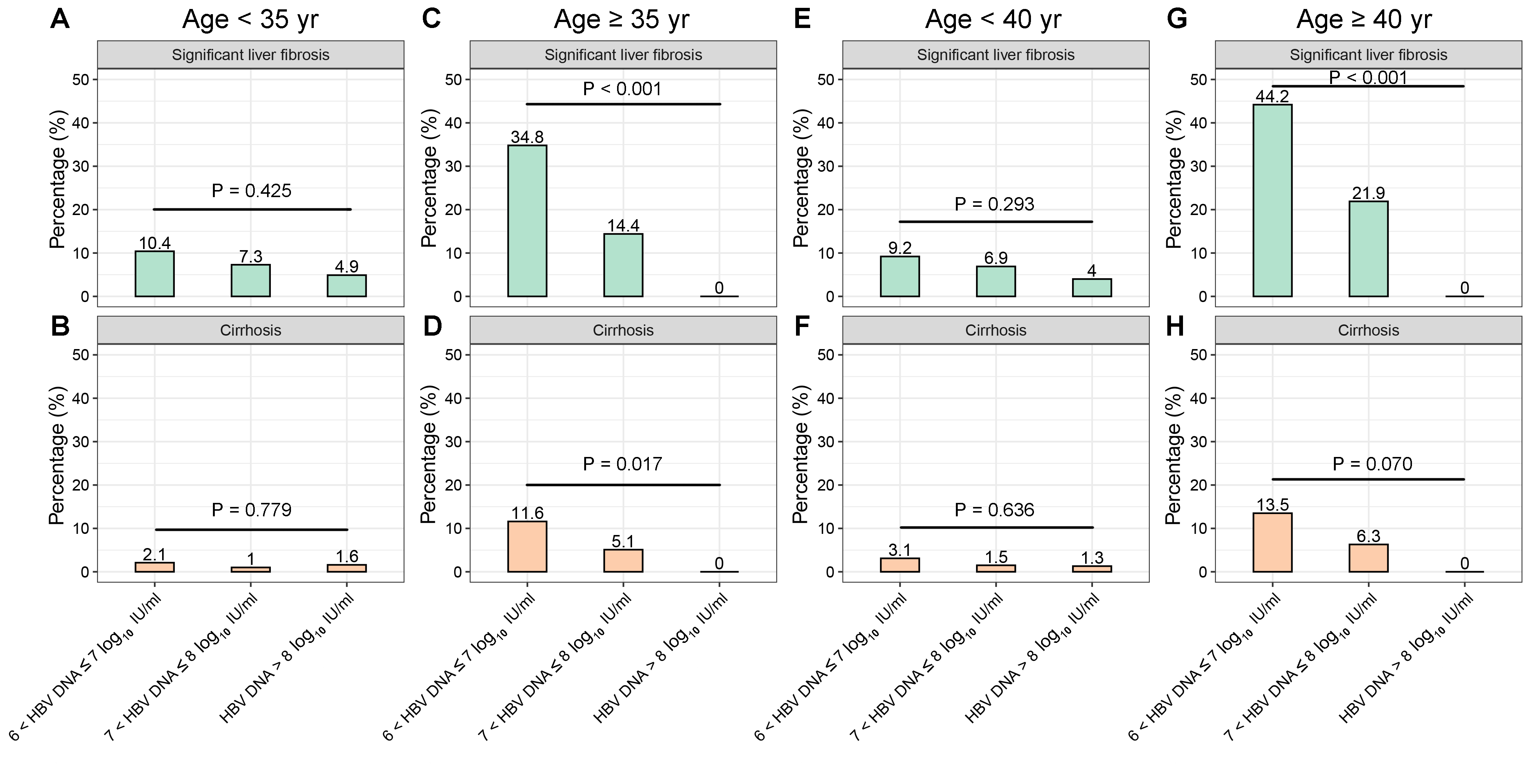

Supplement: Supplementary file 1 — Supplementary Material 1 [file 12985_2024_2368_MOESM1_ESM.docx]
